# The Role of Acromioplasty for Management of Rotator Cuff Problems: Where Is the Evidence?

**DOI:** 10.1155/2012/467571

**Published:** 2012-12-19

**Authors:** Lewis L. Shi, T. Bradley Edwards

**Affiliations:** ^1^Department of Orthopaedics, University of Chicago, 5841 S. Maryland Avenue, MC 3079, Chicago, IL 60637, USA; ^2^Fondren Orthopaedic Group, Texas Orthopedic Hospital, 7401 South Main Street, Houston, TX 77030, USA

## Abstract

The incidence of acromioplasty has increased dramatically in recent decades, but its role in rotator cuff surgery has been debated. Neer popularized the extrinsic theory of rotator cuff pathology, where mechanical compression of the coracoacromial arch leads to tearing of the rotator cuff. Under this theory, acromioplasty is advocated to modify acromial morphology as an essential part of rotator cuff surgery. Proponents of the intrinsic theory suggest rotator cuff tendons undergo degeneration through aging and overuse, and that bursectomy alone without acromioplasty is sufficient. There exist cadaveric studies, expert opinions, and numerous case series espousing both sides of the argument. Recently, however, numerous high-quality prospective randomized controlled trials have been published examining the role of acromioplasty. They have similar study design and randomization protocols, including groups of arthroscopic rotator cuff repair with bursectomy and acromioplasty versus isolated bursectomy. The results have been consistent across all studies, with no difference in the outcomes of the acromioplasty and isolated bursectomy groups. Current evidence does not support the routine use of acromioplasty in the treatment of rotator cuff disease.

## 1. Introduction

Rotator cuff pathology is a spectrum of disease that includes subacromial bursitis, rotator cuff tendinosis, and partial-thickness and full-thickness rotator cuff tear. Neer coined the term impingement syndrome, which has been used to refer to the full range of rotator cuff abnormalities [1]. It is the most commonly diagnosed disorder of the shoulder, accounting for half of all shoulder complaints [2].

Even before Neer, Codman in 1934 described rotator cuff pathology in his landmark book “The Shoulder. Rupture of the Supraspinatus Tendon and Other Lesions in and about the Subacromial Bursa” [3]. He proposed that humeral head-acromion impingement during abduction is the cause of rotator cuff lesions and proposed a lateral acromioplasty. In 1972, Neer suggested that impingement was in fact anterolateral: the anterior acromion, the coracoacromial ligament (CAL), and sometimes inferior acromioclavicular osteophytes [1]. In recent decades, the acromioplasty procedure, as Neer described has become one of the most commonly performed procedures in orthopaedic surgery. Acromioplasty is often performed as part of subacromial decompression, which involves an anteroinferior acromioplasty, CAL release, and subacromial bursectomy [4, 5]. With the advent of arthroscopy, Ellman has popularized the arthroscopic acromioplasty procedure [6]. We review the role of acromioplasty in the management of rotator cuff disorders.

## 2. Extrinsic and Intrinsic Theories

The two models of impingement are the extrinsic or mechanical theory and intrinsic or degenerative theory. In the extrinsic model, espoused by Neer, mechanical compression of the coracoacromial arch leads to the rupture of the rotator cuff [7, 8]. Proponents of this theory advocate acromioplasty to modify acromial morphology as an integral part of rotator cuff surgery [1, 9, 10].

The intrinsic model of impingement suggests that through aging and overuse, rotator cuff tendons undergo degeneration [11, 12]. Bursectomy alone without acromioplasty is considered adequate by those who subscribe to the intrinsic theory, since symptoms are felt to be caused by degenerative tendinopathy and subsequent inflammation of the bursa.

## 3. Rising Incidence of Acromioplasty

The incidence of acromioplasty has increased dramatically in recent decades. Vitale et al. searched two databases to examine trends in frequency of acromioplasty [13]. In the first part of their study, they looked at the New York Statewide Planning and Research Cooperative System ambulatory surgery database from 1996 to 2006. It shows that in this span of 11 years, the incidence of acromioplasty increased from 30.0 to 101.9 per 100,000. The volume of acromioplasty procedures increased at a rate that was 3 times faster than the overall increase of orthopaedic ambulatory procedures. The authors then examined the American Board of Orthopaedic Surgery database from 1999 to 2008. This showed that from 1999 to 2008, the mean number of arthroscopic acromioplasties reported per candidate increased from 2.6 to 6.3, a 142.3% increase, compared to 13.0% increase in the mean number of all orthopaedic procedures. Yu and colleagues from the Mayo clinic also catalogued the rising incidence of anterior acromioplasty using medical records of residents in Olmsted County, Minnesota. The incidence increased from 3.3 per 100,000 from 1980 to 1985 to 19.0 per 100,000 from 2000 to 2005 [14].

## 4. Arguments for and against Acromioplasty

Despite the dramatic increase in the number of acromioplasties being performed, there are numerous arguments for and against this procedure. Potential benefits of acromioplasty include improving coracoacromial arch anatomy to reduce extrinsic compression on the rotator cuff, improved arthroscopic visualization during rotator cuff repair, and inducing a healing response through bleeding bone in the subacromial space [1, 9, 10].

The arguments against acromioplasty include preservation of the CAL and deltoid attachment, the economics of saved operative time and equipment, and the neurobiology of subacromial space.

- Codman admonished in 1934 that “the coracoacromial arch has an important duty and should not be thoughtlessly divided at any operation” [3]. With acromioplasty, insertion of the CAL can be compromised, if not intentionally released. In the setting of irreparable rotator cuff tear, patients may experience anterosuperior instability of the humeral head [15]. Fagelman et al. even go as far as recommending reconstruction of the CAL after previous rotator cuff surgery and acromioplasty, in an attempt to prevent anterosuperior escape [16].
- In addition to CAL injury, acromioplasty can lead to deltoid detachment. In a cadaveric study, Green et al. showed that a 4 mm of resection of bone from the undersurface of the acromion resulted in release of 56% (±11%) of deltoid origin, and resection of 5.5 mm of bone resulted in release of 77% (±15%) of deltoid origin [17].
- There are no reports directly comparing cost effectiveness of rotator cuff surgery with and without acromioplasty; however, it is obvious that there are time and equipment costs associated with this procedure.
- In treating pain associated with rotator cuff disease, subacromial bursectomy alone may be effective. This can be explained by evidence suggesting that an inflamed and thickened bursa is the pain generator in impingement syndrome, and that removal of the bursa would provide pain relief [18, 19].

## 5. Evidence for and against Acromioplasty

With the arguments for and against acromioplasty based on cadaveric studies and expert-level opinions, numerous investigators have used evidence-based methods to examine the role of acromioplasty in rotator cuff surgery.

### 5.1. Nonoperative Treatment versus Acromioplasty

Ketola and colleagues performed a randomized controlled trial comparing supervised therapy to therapy plus arthroscopic acromioplasty in the treatment of shoulder impingement syndrome [20]. They enrolled 70 patients in each arm and had a follow-up rate of 96%. The primary outcome measure evaluated was improvement in pain visual analog score (VAS) between before surgery and 24 months postoperatively. No differences in outcomes were observed between the treatment groups. The authors concluded that acromioplasty provides no clinically important benefits compared to an exercise program in terms of subjective outcome or cost-effectiveness.

### 5.2. Bursectomy Alone versus Bursectomy with Acromioplasty: Lower Level Evidence

Acromioplasty has been advocated as an integral part of rotator cuff repair. Gartsman et al. performed arthroscopic acromioplasty with rotator cuff repair, after which the mean University of California Los Angeles (UCLA) score, American Shoulder and Elbow Surgeons (ASES) shoulder score, and Constant score all improved significantly at 30-month followup [21]. Blevins et al. reported on the outcome of 64 patients who underwent mini-open cuff repair that included an arthroscopic acromioplasty and resection of the CAL [22]. They were followed for 29.2 months, and postoperatively there was a significant increase in active elevation and strength. There was an improvement in pain score, and 89% of the patients were satisfied.

Other investigators have published case series highlighting the success of rotator cuff repair without acromioplasty. McCallister et al. reported on 61 patients who underwent repair of full-thickness rotator cuff tears with preservation of the integrity of the coracoacromial arch and deltoid insertion [23]. They were followed for an average of 5 years, and patients had significant improvement in their Simple Shoulder Test scores as well as health-related qualify of life measurements (SF-36). Budoff et al. also reported good and excellent results in 81% of patients at medium-term follow-up of 5 years in patients being treated for partial-thickness rotator cuff tears with only bursectomy and no acromioplasty [24]. A follow-up study of the same cohort reported 79% good or excellent results at 9.5 years postoperatively [25].

All of these studies are level 4 evidence because of lack of comparison groups.

### 5.3. Bursectomy Alone versus Bursectomy with Acromioplasty: Higher Level Evidence

In recent years, several high-quality prospective randomized controlled trials have been published to investigate the role of acromioplasty in the treatment of rotator cuff disease.  Table 1 summarizes these studies' designs and pertinent results.

Henkus et al. published a randomized controlled trial focusing on the treatment of impingement syndrome without rotator cuff tears [26]. Fifty-seven patients were randomized either into arthroscopic bursectomy (26 patients) or bursectomy with acromioplasty groups (30 patients); one patient was lost to follow up. At a mean follow-up of 2.5 years (range, 1–5 years), both bursectomy and acromioplasty groups had good clinical outcomes, and there were no significant differences between the groups. Acromial morphology affected the outcome of both groups, but when stratified based on acromial morphology, the two groups again were no different in outcome.

There are four other prospective randomized controlled trials which enrolled patients with full-thickness rotator cuff tears, with some studies using acromial morphology as an additional inclusion criteria. They all had similar randomization design with one group having arthroscopic rotator cuff repair with subacromial bursectomy and acromioplasty (ARCR-A) and another group having arthroscopic rotator cuff repair with bursectomy alone (ARCR). Shin et al. focused on small (<1 cm) and medium-sized (1–3 cm) full-thickness rotator cuff tears [27]. Accounting for a follow-up rate of 80%, the authors enrolled 60 patients in each arm of the study and followed them for 35 months postoperatively. Using Constant score, ASES shoulder score, UCLA score, and pain VAS, there was no significant difference between the two groups. All patients had postoperative magnetic resonance imaging, and the retear rates were similar in both groups: 17% in ARCR-A and 20% in ARCR (*P* = 0.48). The paper concluded that acromioplasty may not be necessary in the operative treatment of patients with small- to medium-sized rotator cuff tears.

MacDonald et al. enrolled patients with full-thickness cuff tears of the supraspinatus and/or infraspinatus, and all acromial morphologies were included [28]. Eighty-six patients were enrolled in the study, and 68 patients were available at minimum 24-month followup. Western Ontario Rotator Cuff (WORC) index and ASES shoulder scores were not different between ARCR-A and ARCR groups. There were no differences in patients' outcomes based on acromial type, nor were there any interactive effects identified between treatment group and acromial type. Four patients (9%) in ARCR group required revision surgery (1 with a type-2 acromion and 3 with type-3 acromions) compared to no patients in the ARCR-A group (*P* = 0.05). The authors emphasized that the decision to undergo revision surgery was based on patient symptoms and not on postoperative imaging. They concluded that it was possible that the higher reoperation rate, including recurrent cuff tears, occurred as result of unaltered acromial morphology in the ARCR group.

Milano et al. enrolled 80 patients with full-thickness rotator cuff tear with type-2 and-3 acromions [29]. There was 89% followup at 24 months, with 34 patients in the ARCR-A group and 37 patients in the ARCR group. Acromioplasty did not influence clinical outcome, as measured by Constant score, DASH, and work-DASH.

Gartsman and O'Connor included only patients with reparable full-thickness tears of the supraspinatus and type-2 acromions [30]. Ninety-three patients were randomized and followed for a mean of 15.6 months, shorter than other studies. The ASES shoulder score was the only outcome measurement, and there was no significant difference between the ARCR-A and the ARCR groups. The strengths of this study include well-defined inclusion criteria, single surgeon, and a follow-up rate of 100%.

## 6. Conclusion

There is increasing number of published reports examining the role of acromioplasty in the treatment of rotator cuff disease. On the basis of the current literature, patients have similar outcome independent of whether or not an acromioplasty is performed at short and intermediate followup, regardless of acromion morphology. The notable exception is that MacDonald reported a higher rate of reoperation at 24 months in the non acromioplasty group (*P* = 0.05) [28]. The nonblinded nature of this study calls into question the validity of reoperation rate as a secondary outcome measure, a point the authors acknowledged.

Pedowitz et al. recently published rotator cuff disease treatment guidelines established by the American Academy of Orthopaedic Surgeons [31]. They suggested that “routine acromioplasty is not required at the time of rotator cuff repair,” with a “moderate” grade of recommendation. That report based its guidelines on the two prospective randomized controlled trials available at that time by Milano et al. and Gartsman and O'Connor [29, 30]. With subsequent reports reinforcing these findings, recommendations may be upgraded to “strong.”

With multiple well-designed studies suggesting acromioplasty providing no benefits in terms of pain relief, function or quality of life, evidence does not support the routine use of acromioplasty in the treatment of rotator cuff disease.

## Figures and Tables

**Table 1:** Summary of prospective randomized controlled trials studying the role of acromioplasty in treatment of rotator cuff disease.

Author	Journal/year	Inclusion criteria	Patients/age	Follow up rate and length	Randomization	Measured outcomes	Primary result	Secondary result
Henkus et al. [26]	JBJS-B 2009	Subacromial impingement syndrome	57 pts; mean age 47	56/57 (98%); 30 months	26 bursectomy only; 30 bursectomy w/acromioplasty	Constant, SST, pain VAS	No difference	Acromion morphology made no difference in outcome

Shin et al. [27]	Arthroscopy 2012	Small-to medium-sized rotator cuff tears	150 pts; mean age 56.8	120/150 (80%); 35 months	60 in ARCR-A; 60 in ARCR	Constant, ASES, UCLA, pain VAS	No difference	Acromion morphology made no difference in outcome; retear rates (MRI): 17% ARCR-A versus 20% ARCR (*P* = 0.48)

MacDonald et al. [28]	JBJS-A 2011	Full-thickness cuff tears;	86 pts; mean age 56.8	68/86 (79%); 24 months	32 in ARCR-A; 36 in ARCR	WORC, ASES	No difference	Revision rate: 0/32 ARCR-A versus 4/36 in ARCR (*P* = 0.05)

Milano et al. [29]	Arthroscopy 2007	Full-thickness cuff tears; type 2 and 3 acromions	80 pts; mean age 60.4	71/80 (89%); 24 months	34 in ARCR-A; 37 in ARCR	Constant, DASH, work-DASH	No difference	

Gartsman and O'Connor [30]	JSES 2004	Full-thickness supraspinatus tear, type 2 acromion	93 pts; mean age 59.7	93/93 (100%); 15.6 months	47 in ARCR-A; 46 in ARCR	ASES	No difference	

ARCR-A: arthroscopic rotator cuff repair with bursectomy and acromioplasty; ARCR: arthroscopic rotator cuff repair with bursectomy only; SST: Simple Shoulder Test score; VAS: visual analog score; UCLA: University of California Los Angeles Shoulder score; WORC: Western Ontario Rotator Cuff index; ASES: American Shoulder and Elbow Society shoulder score; DASH: disability of arm, shoulder and hand score.
